# Case Report: Craniofacial deafness hand syndrome with unusual cardiovascular symptoms and lack of holistic care

**DOI:** 10.3389/fgene.2024.1354632

**Published:** 2025-01-07

**Authors:** Samantha Saenz Hinojosa, Carlos Reyes-Silva, Kazuyoshi Hosomichi, Vanessa I. Romero

**Affiliations:** ^1^ Human Genetics Department, School of Medicine, Universidad San Francisco de Quito, Quito, Ecuador; ^2^ Genetics Department, Hospital de Especialidades Eugenio Espejo, Quito, Ecuador; ^3^ Laboratory of Computational Genomics Department, Tokyo University of Pharmacy and Life Sciences, Tokyo, Japan

**Keywords:** PAX3 variation, pulmonary hypertension, patent ductus arteriosus, pathogenic variant, Ecuador

## Abstract

**Background:**

Delays in diagnosing rare genetic disorders often arise due to limited awareness and systemic challenges in primary care. This case highlights the importance of a holistic approach to patient care, encompassing timely detection and comprehensive evaluation of clinical features.

**Methods:**

We report the case of a 21-year-old Ecuadorian male with facial and hand dysmorphias, cardiomegaly, pulmonary hypertension, and patent ductus arteriosus (PDA). Whole-exome sequencing, performed using the Illumina NextSeq platform. We extensively analyzed over 100 genes linked to congenital structural heart diseases.

**Results:**

The genetic findings provided a definitive diagnosis of Craniofacial-Deafness-Hand Syndrome, an extremely rare autosomal dominant condition, but found no variants that explain the patient’s cardiac phenotype. We identified a novel pathogenic missense variant in the *PAX3* gene (c.A91C, p. T31P).

**Discussion and conclusions:**

This case underscores the necessity of integrating genetic testing into routine clinical practice to enhance diagnostic precision for rare diseases. It also highlights the need for multidisciplinary collaboration and a holistic care model to improve patient outcomes. The unique association of Craniofacial-Deafness-Hand Syndrome with cardiovascular anomalies due to a *PAX3* variation provides valuable insights into the genetic underpinnings of this rare condition.

## Introduction

The diagnosis of patients with rare disorders often encounters significant delays due to various factors. Patients typically undergo multiple consultations with medical professionals from different specialties before seeking genetic appointments. One reason for this delay is the limited familiarity or lack of clinical exposure to geneticists among primary care physicians (Haga et al., 2013). Moreover, primary care physicians may not prioritize genetic referrals due to factors such as limited knowledge, lower socioeconomic status of patients, and rigid administrative structures (Fok et al., 2021, p. 1). Consequently, the diagnostic journey for patients with rare disorders can span several years, with studies reporting diagnostic delays ranging from 1 to 10 years or more (Gahl and Tifft, 2011).

Craniofacial-Deafness-Hand Syndrome (CDHS) is an extremely rare autosomal dominant disorder characterized by distinctive facial dysmorphisms, sensorineural hearing loss, and hand anomalies, often presenting with features such as narrow nostrils, hypoplastic ears, and clinodactyly. Variations in the *PAX3* gene have been associated with various conditions, including Waardenburg syndrome, alveolar rhabdomyosarcoma, and CDHS (Birrane et al., 2009). The *PAX3* gene is located on the long arm of chromosome 2 and encodes a transcription factor belonging to the PAX family. These transcription factors are characterized by a highly conserved paired box motif (Boudjadi et al., 2018). The PAX3 protein consists of an N-terminal DNA binding domain and a C-terminal transcriptional activation domain (Boudjadi et al., 2018). During development, this protein is expressed in skeletal muscle, the central nervous system, and neural crest derivatives. It plays a crucial role in regulating the expression of target genes that influence proliferation, survival, differentiation, and motility in these lineages (Boudjadi et al., 2018).

We present the case of a 21-year-old male with CDHS who exhibited facial dysmorphias, hand abnormalities, bilateral sensorineural hearing loss, and cardiovascular complications, including severe pulmonary hypertension and patent ductus arteriosus (PDA). Whole-exome sequencing identified a novel pathogenic missense variant in the *PAX3* gene (c.A91C, p. T31P), which is causative of CDHS in this patient. The delayed diagnosis highlights the critical need for early genetic testing and a holistic approach to patient care.

## Case presentation and diagnostic assessment

**FIGURE 1:**
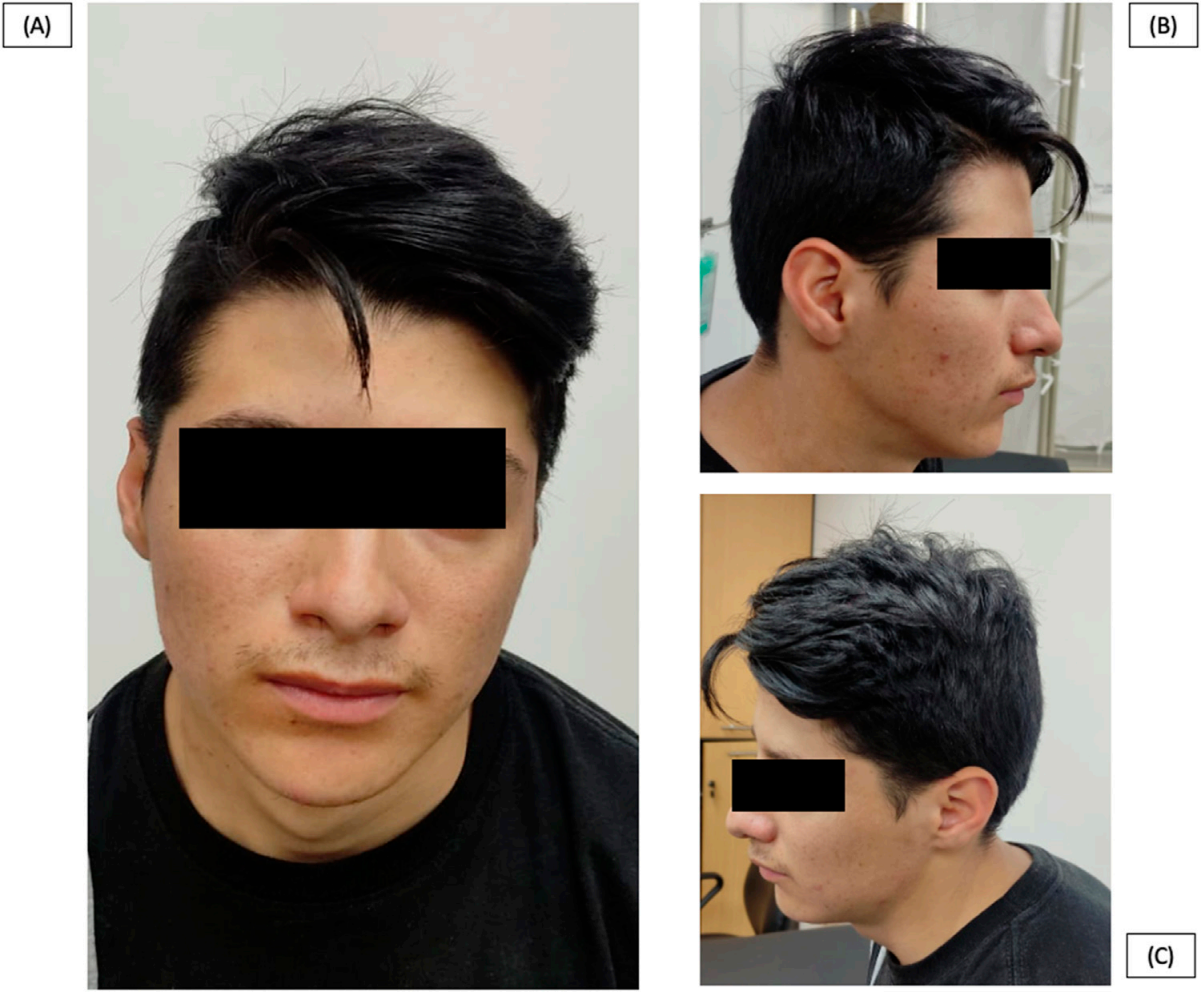
Patient at 21 years old. **(A)**, **(B)**, **(C)** Facial asymmetry, narrow nostrils, and small ears.

**FIGURE 2:**
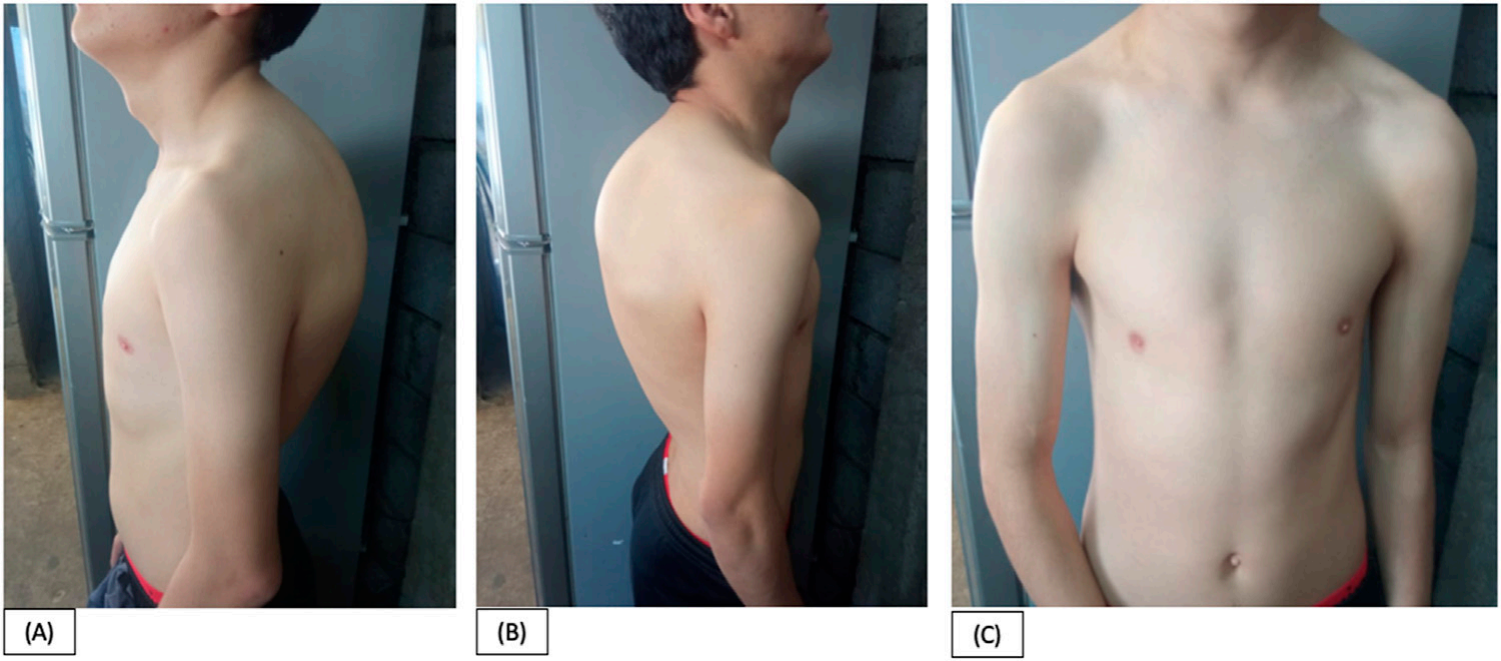
Patient at 21 years old. **(A)**, **(B)**, **(C)**. Asymmetrical thorax with hypoplasia of the shoulder girdle, kyphoscoliosis, and pectus carinatum.

**FIGURE 3:**
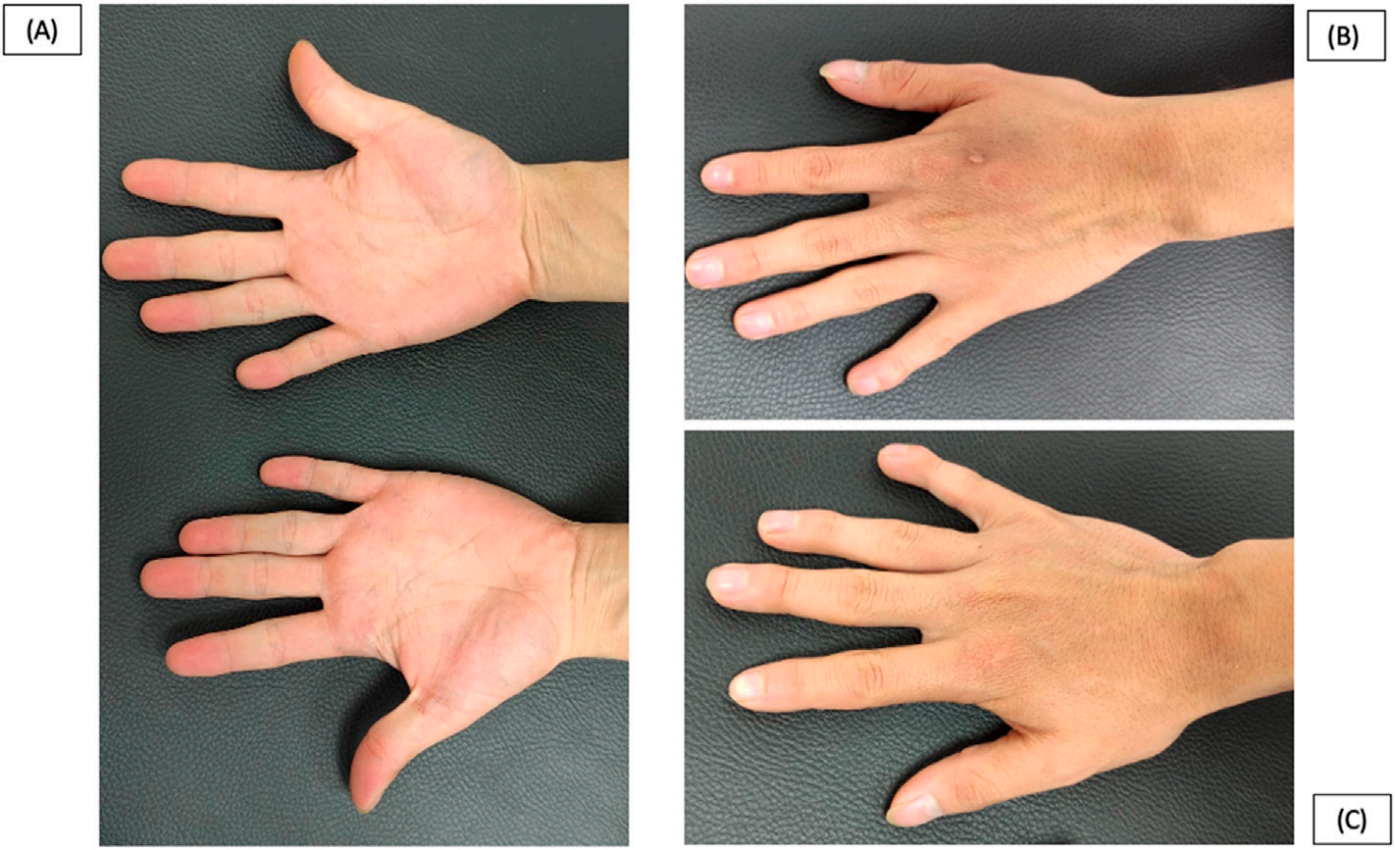
Patient at 21 years old. **(A)**, **(B)**, **(C)**. Nail clubbing, increased distal interphalangeal joints in the fifth finger and proximal in second and third fingers bilaterally and shortening of the fifth finger and clinodactyly.

We present a clinical case involving a 21-year-old Ecuadorian male who was referred to our genetic outpatient clinic due to a combination of facial and hand dysmorphias alongside a cardiopathy. At the age of 19-years, the patient experienced dyspnea upon exertion, leading to a diagnosis of an untreatable PDA by a cardiologist. Notably, a physical examination revealed below-average height for his age (158 cm). Regarding facial characteristics, the patient exhibited microcephaly (head circumference of 50 cm), asymmetry, a hypopigmented lesion in the right iris, narrow nostrils, and small ears (Figure 1). Additionally, the thorax displayed asymmetry with hypoplasia of the shoulder girdle, kyphoscoliosis, and pectus carinatum (Figure 2). Hand abnormalities included nail clubbing, increased distal interphalangeal joints in the fifth finger, and proximal joints in the second and third fingers bilaterally, as well as shortening and clinodactyly of the fifth finger. Hyperkinetic pulses were observed, along with bilateral cubitus valgus in the feet (Figure 3). At infancy, the patient’s pediatrician noted the presence of a heart murmur without providing further details or follow-up. During cardiac examination, an ejection systolic murmur of grade III/VI and a diastolic murmur of grade IV/VI were detected. Furthermore, the patient reported hearing loss that originated at the age of 8-years, and subsequent otoscopic examination revealed moderate to severe neurosensorial hearing loss in the right ear and profound neurosensorial hearing loss in the left ear. Importantly, neither the parents nor the family reported the presence of similar clinical presentations to the patient among other family members.

**FIGURE 4:**
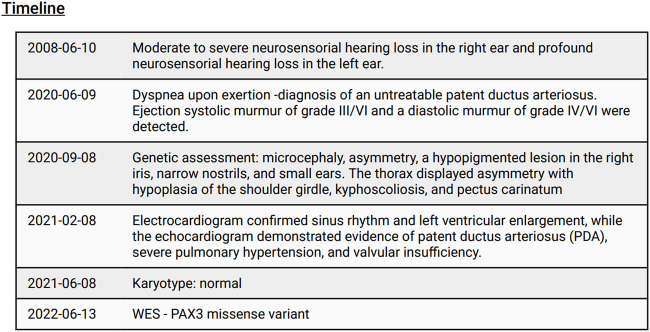
CARE timeline.

The chest x-ray revealed grade II cardiomegaly and indications of pulmonary hypertension. The electrocardiogram confirmed sinus rhythm and left ventricular enlargement, while the echocardiogram demonstrated evidence of PDA, severe pulmonary hypertension, and valvular insufficiency. Considering the presence of pulmonary hypertension, the cardiologist determined that surgical repair of the PDA was not a viable option for the patient. As a result, symptomatic treatment was prescribed, which included a loop diuretic (furosemide), a dual endothelin receptor antagonist (bosentan), an angiotensin-converting-enzyme inhibitor (enalapril), and a phosphodiesterase inhibitor (sildenafil). These medications aim to manage symptoms rather than provide curative treatment (Figure 4).

The patient’s karyotype analysis produced normal results. To investigate the underlying genetic condition, whole exome sequencing (WES) was performed on the patient’s peripheral blood using the Illumina NextSeq1000 platform with a protocol involving probe-based capture of target regions followed by next-generation sequencing. A total of 41,660,733 reads were obtained, with 99.86% successfully mapped (41,603,016 reads). Raw data were aligned using the Burrows–Wheeler Aligner, and sorting and merging were carried out with Picard tools. Variant calling for single nucleotide variants and insertions/deletions was conducted with GATK version 4.3, using the GRCh37 human genome as a reference. Variants were annotated with ANNOVAR, integrating functional prediction algorithms including SIFT, PolyPhen-2, MutationTaster, PROVEAN, and FATHMM, alongside databases such as gnomAD, which confirmed the variant’s absence in the general population.

A total of 949 exonic nonsynonymous variants were identified, excluding synonymous changes. Among these, we identified a novel heterozygous c. A91C missense variant in exon 2 of the *PAX3* gene (HGNC: 8617, NM: 000438), resulting in a threonine-to-methionine substitution at position 31 (p.T31P). Predictive algorithms indicated this variant was pathogenic, with scores such as SIFT (0.015, damaging), PolyPhen-2 (1, deleterious), MutationTaster (1, disease-causing), PROVEAN (−3.13, deleterious), and FATHMM (−3.43, deleterious). Conservation analysis showed the threonine residue is highly conserved across species, including zebrafish, suggesting a significant impact on protein function. Sanger sequencing confirmed the presence of this pathogenic variant, and further analysis excluded other genetic causes of the patient’s cardiopathy.

The patient’s variant is found within the conserved paired box domain (PB) (Supplementary Figure S1), consisting of two subdomains required for all isoforms. Each subdomain adopts a three-helix fold with a helix-turn-helix (HTH) motif. The C-terminal helix of the HTH interacts with specific DNA bases in the major groove. The PB can identify similar DNA sequences (TCACGC/G), although minor variations are observed among different PAX orthologues (Boudjadi et al., 2018). Following the Standards and Guidelines for the Interpretation of Sequence Variants the variant can be classified as pathogenic as there is no family history (PS2, PM6), is absent in the publicly available population databases (PS4, PM2), it is located in a well-established functional domain (PM1), is located in an evolutionary conserved aminoacid (PM4), *in silico* programs agreed in a damaging prediction (PP3), and the patient has well defined clinical characteristics (PP4) (Richards et al., 2015). Furthermore, we conducted an extensive search for variations in more than 100 genes associated with congenital structural heart diseases. However, the patient’s cardiac phenotype does not appear to be caused by any variants in these genes (Supplementary Table S1). This additional analysis helps to exclude other genetic causes specifically related to congenital structural heart diseases as contributing factors to the patient’s cardiovascular malformation.

## Discussion and conclusion

We present the case of a 21-year-old male patient who initially presented with a heart murmur at the age of 5 months. Unfortunately, no medical follow-up was pursued until he reached 19 years of age, when he began experiencing symptoms and sought consultation from various specialists, including cardiology, ophthalmology (no colobomas or retinal dystrophy), otorhinolaryngology (bilateral sensorineural hearing loss, moderate-severe on the right and profound on the left), and nutrition (undernutrition). Despite these consultations, a comprehensive diagnosis was not achieved, as each specialist focused solely on their respective areas of expertise. This diagnostic fragmentation delayed recognition of a syndromic condition that could have been identified earlier. Eventually, the patient was referred to a geneticist due to the presence of facial dysmorphisms, which are classical features of many syndromes and can include microcephaly, low-set ears, hypotelorism or hypertelorism, micrognathia or retrognathia, frontal bossing, and a sloping forehead (Eixarch, et al.,2018). The patient exhibited additional features such as microcephaly, hand dysmorphia, and hearing loss, further complicating the diagnosis process. This case underscores the importance of early genetics referral in patients presenting with dysmorphic features and complex medical issues to prevent diagnostic delays.

To overcome these challenges, a comprehensive diagnostic approach was employed. WES analysis was performed, which revealed a variation in the *PAX3* gene (Wang et al., 2008). This genetic finding provided crucial insights into the underlying cause of the patient’s condition. The presented case highlights the significance of interdisciplinary collaboration and the involvement of genetic specialists in the diagnostic process. In complex cases with overlapping symptoms and manifestations, such collaboration is essential to ensure accurate and comprehensive assessments, leading to an appropriate diagnosis. The extensive search for variations in over 100 genes associated with congenital structural heart diseases underscores the thorough approach taken to investigate the genetic basis of the patient’s cardiovascular malformation. The absence of pathogenic variants in these genes (Supplementary Table S1) supports the exclusion of established genetic causes linked to these conditions.

The craniofacial-deafness-hand syndrome is an extremely rare autosomal dominant disorder with a prevalence of less than 1 in 1,000,000, and it is diagnosed in our patient (Gad et al., 2008). Typically, symptoms of this syndrome manifest in newborns between birth and 4 weeks of age (Gad et al., 2008). In 1983, Sommer et al. first described a 26-year-old mother and her newborn daughter with dysmorphic facial features and sensorineural hearing loss, leading to the diagnosis of craniofacial-deafness-hand syndrome (Sommer et al., 1983). In 2003, Sommer et al. provided a follow-up on these patients and reported a new case: a boy born 2 years after the first daughter, exhibiting similar manifestations (Sommer and Bartholomew, 2003). In 2008, Gad et al. reported a case of a 37-year-old woman with bilateral sensorineural hearing loss and facial abnormalities (Gad et al., 2008). Notably, none of these patients were described as having an intellectual disability.

In 1990, Mathieu et al. reported a case of Waardenburg syndrome type I, which is also associated with *PAX3* variations, and the patient exhibited severe congenital heart disease (Mathieu et al., 1990). This finding prompted us to investigate whether there is a potential connection between the variation in our patient and his cardiovascular symptoms. Interestingly, the mouse model Splotch, which carries a variation in the *PAX3* gene, displays cardiac phenotypes such as myocardial dysfunction, persistent truncus arteriosus, and outflow malalignment, among others (Hutson and Kirby, 2007). The *PAX3* gene is highly expressed in the dorsal neural tube and migrating cardiac neural crest cells (Hutson and Kirby, 2007). Steele et al. reported that genetic alterations in the *PAX3* gene in mouse models lead to defects associated with neural crest derivatives and cardiovascular malformations (Steele et al., 2022). Notably, our patient represents the first reported case of Craniofacial-Deafness-Hand Syndrome associated with a *PAX3* variation and PDA, highlighting the gene’s role in craniofacial, auditory, hand, and cardiovascular development. The diagnosis was confirmed by the patient’s clinical features and a novel pathogenic *PAX3* variant (c.A91C, p. T31P) in a conserved functional domain, meeting ACMG criteria and aligning with the phenotype.

This case highlights two key lessons: the necessity of a holistic patient evaluation to reduce complications and the importance of genetic testing in rare diseases. Holistic care emphasizes comprehensive assessment, accurate diagnosis, appropriate treatment, and follow-up. In this case, the patient’s heart murmur, likely linked to a PDA, was detected early but left untreated. PDA, characterized by a medium-pitched continuous murmur, may stem from factors such as developmental immaturity or structural abnormalities involving neural crest cell derivatives or fibronectin-dependent smooth muscle cell migration (Backes et al., 2022; Schneider and Moore, 2006). Although initially asymptomatic, PDA can cause complications like heart failure, infective endocarditis, and pulmonary hypertension if untreated (Schneider and Moore, 2006). Treatment options include pharmaceutical closure with indomethacin or ibuprofen and, if necessary, surgical or catheter-based intervention. A holistic, multidisciplinary approach addresses physical, mental, social, and spiritual wellbeing, improving patient outcomes and health equity (Tsai and Yan, 2021; Garcia, 2022; Rubin et al., 2016). The second lesson underscores the value of genetic testing in diagnosing rare diseases. Collaboration among general physicians, pediatricians, and geneticists is crucial for molecular testing, which establishes a definitive diagnosis and guides management (Mori et al. 2024). While treatments for some rare diseases remain unavailable, accurate diagnosis supports patients and families and informs research into new symptoms and management strategies (Borozdin et al., 2006; Retterer et al., 2016). Genetic testing, particularly WES, is vital for diagnosing genetically heterogeneous disorders and can identify co-occurring conditions when clinical presentations are unclear. WES expedites diagnosis, reduces costs, and minimizes unnecessary procedures, and ongoing research explores its integration into newborn screening. However, challenges such as cost, privacy, access, and informed consent persist, particularly in regions like Latin America, where testing availability is limited, forcing patients to seek expensive services abroad. Early diagnosis through genetic testing significantly enhances patient quality of life, providing clarity and improving care.

We have presented a case report detailing a novel nonsynonymous variation in the *PAX3* gene which can be classified as pathogenic, resulting in an atypical manifestation of Craniofacial-Deafness-Hand Syndrome accompanied by a cardiovascular defect. This review underscores the importance of treating patients holistically and considering their entire clinical presentation. Patients rely on physicians to listen to their stories and symptoms in order to provide effective care. The patient described in this report serves as a poignant example of the consequences that can arise from failing to consider all relevant characteristics and symptoms, leading to a delayed diagnosis and numerous complications with limited treatment options. It is imperative for physicians to engage in interdisciplinary collaboration, seeking input from various specialists, and employing diverse diagnostic tools, including genetic testing, to ensure accurate and comprehensive diagnoses. By adopting this approach, healthcare professionals can provide more effective and timely care, thereby improving patient outcomes.

## Data Availability

The original contributions presented in the study are included in the article/Supplementary Materials, further inquiries can be directed to the corresponding author

## Abbreviations

PDA, Patent Ductus Arteriosus; WES, Whole Exome Sequencing; DNA, Deoxyribonucleic acid
