# Supplementary figures and images for: Case Report: Craniofacial deafness hand syndrome with unusual cardiovascular symptoms and lack of holistic care
Source: Front Genet. 2025 Jan 7;15:1354632. doi: 10.3389/fgene.2024.1354632 (PMC11754966; doi:10.3389/fgene.2024.1354632)

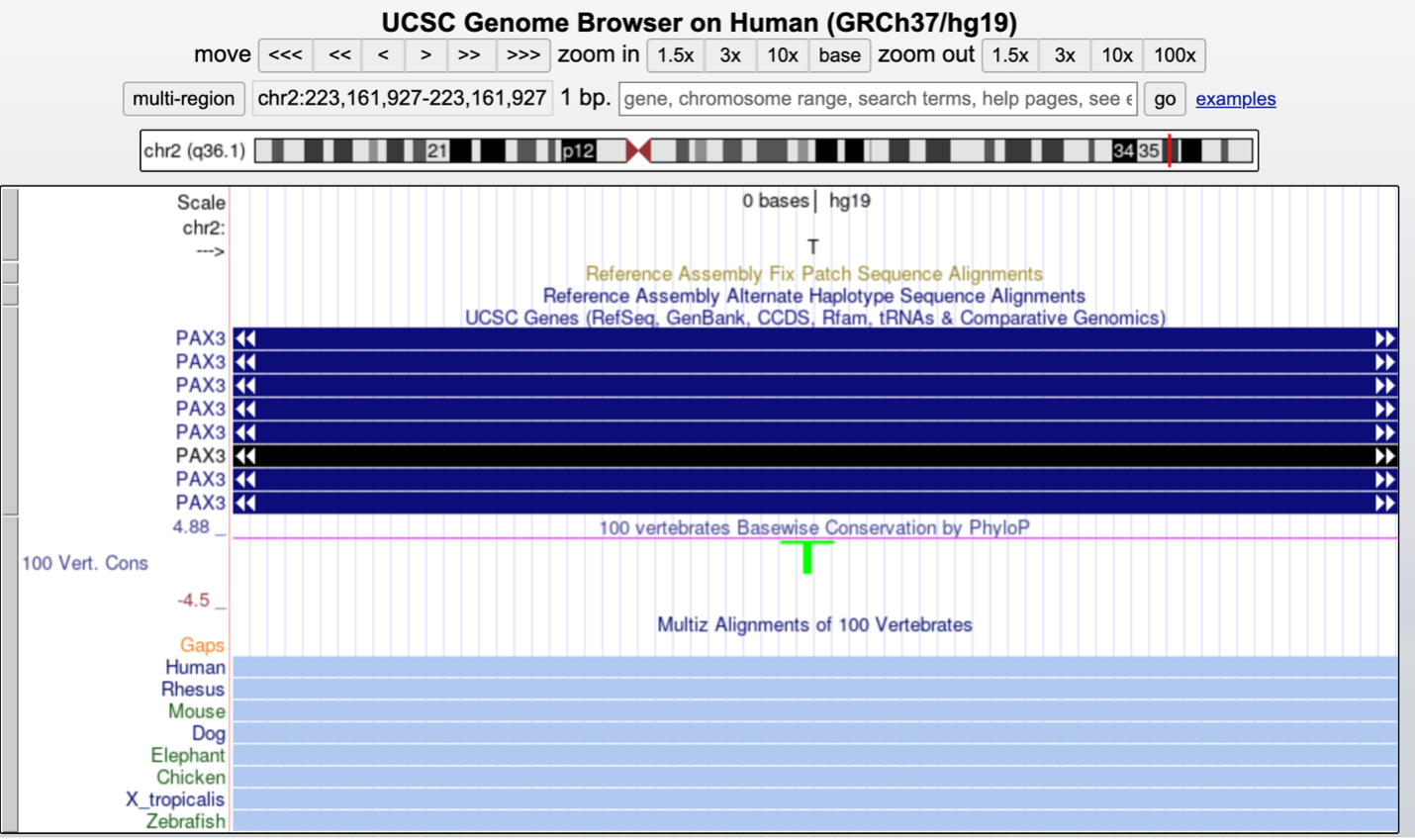

Supplement: Supplementary file 2 [file Image1.png]
